# Beta-3 adrenergic agonists reduce pulmonary vascular resistance and improve right ventricular performance in a porcine model of chronic pulmonary hypertension

**DOI:** 10.1007/s00395-016-0567-0

**Published:** 2016-06-21

**Authors:** Ana García-Álvarez, Daniel Pereda, Inés García-Lunar, David Sanz-Rosa, Rodrigo Fernández-Jiménez, Jaime García-Prieto, Mario Nuño-Ayala, Federico Sierra, Evelyn Santiago, Elena Sandoval, Paula Campelos, Jaume Agüero, Gonzalo Pizarro, Víctor I. Peinado, Leticia Fernández-Friera, José M. García-Ruiz, Joan A. Barberá, Manuel Castellá, Manel Sabaté, Valentín Fuster, Borja Ibañez

**Affiliations:** Centro Nacional de Investigaciones Cardiovasculares Carlos III (CNIC), Madrid, Spain; Hospital Clínic, IDIBAPS, Barcelona, Spain; Hospital Universitario Quirón Madrid, UEM, Madrid, Spain; Hospital Clínico San Carlos, Madrid, Spain; Centro de Investigación Biomédica en Red (CIBER) de Enfermedades Respiratorias, Barcelona, Spain; Hospital Universitario Montepríncipe, Madrid, Spain; Hospital Universitario Central de Asturias, Oviedo, Spain; Zena and Michael A. Wiener Cardiovascular Institute, Mount Sinai School of Medicine, New York, USA; Department of Cardiology, IIS-Fundación Jiménez Díaz, Madrid, Spain

**Keywords:** Beta-3 adrenergic receptor, Pulmonary hypertension, Therapy, Pulmonary vascular resistance

## Abstract

Beta-3 adrenergic receptor (β3AR) agonists have been shown to produce vasodilation and prevention of ventricular remodeling in different conditions. Given that these biological functions are critical in pulmonary hypertension (PH), we aimed to demonstrate a beneficial effect of β3AR agonists in PH. An experimental study in pigs (*n* = 34) with chronic PH created by pulmonary vein banding was designed to evaluate the acute hemodynamic effect and the long-term effect of β3AR agonists on hemodynamics, vascular remodeling and RV performance in chronic PH. Ex vivo human experiments were performed to explore the expression of β3AR mRNA and the vasodilator response of β3AR agonists in pulmonary arteries. Single intravenous administration of the β3AR agonist BRL37344 produced a significant acute reduction in PVR, and two-weeks treatment with two different β3AR selective agonists, intravenous BRL37344 or oral mirabegron, resulted in a significant reduction in PVR (median of −2.0 Wood units/m^2^ for BRL37344 vs. +1.5 for vehicle, *p* = 0.04; and −1.8 Wood units/m^2^ for mirabegron vs. +1.6 for vehicle, *p* = 0.002) associated with a significant improvement in magnetic resonance-measured RV performance. Histological markers of pulmonary vascular proliferation (p27 and Ki67) were significantly attenuated in β3AR agonists-treated pigs. β3AR was expressed in human pulmonary arteries and β3AR agonists produced vasodilatation. β3AR agonists produced a significant reduction in PVR and improved RV performance in experimental PH, emerging as a potential novel approach for treating patients with chronic PH.

## Introduction

Pulmonary hypertension (PH) comprises a group of diseases characterized by a chronic increase in pulmonary arterial pressure (PAP) and pulmonary vascular resistance (PVR), and progressive right ventricular (RV) dysfunction [16]. Globally, prevalence of PH is high as it affects nearly 50 % of patients with heart failure [37] or chronic obstructive pulmonary disease [1], and it is associated with a poor prognosis [21]. Few therapies are currently available for pulmonary arterial hypertension (group 1 in the current PH classification), but no pharmacological therapy has been demonstrated to have a consistent effect in PH due to left heart disease (group 2), which is the most frequent cause of PH [15, 37]. Right heart catheterization remains mandatory for the precise quantification of pulmonary hemodynamics [21] and, while diagnosis of PH is currently based on mean PAP, PVR provides a more consistent prognostic value in PH of different etiologies [6, 32].

Impairment of nitric oxide synthesis and signaling through the soluble guanylate cyclase-cyclic guanylate monophosphate pathway [17, 24] are implicated in the pathogenesis of PH causing vasoconstriction and structural remodeling. Therapies that increase nitric oxide release and guanylate cyclase-cyclic guanylate monophosphate availability, in addition to their vasodilator effect, have an antiproliferative effect on the pulmonary vasculature [35, 38].

Beta-3 adrenergic receptor (β3ARs) mRNA expression has been found in the human myocardium [23] and vessels [8], and it has been described to be upregulated in left heart disease [23], although convincing evidence of functional β3ARs in the human heart is still a matter of debate [36]. Recently, several experimental studies have shown that in vivo treatment with BRL37344, a β3AR agonist, improves cardiac performance and ameliorates myocardial injury in experimental models of heart failure [5, 25] and ischemia–reperfusion [2, 14, 26] through a nitric oxide-mediated mechanism. In pulmonary vessels from dogs [33] and rats [10], ex vivo β3AR agonists produces vasodilatation. No studies evaluating the effect of β3AR in PH have been conducted.

We therefore, hypothesized that β3AR agonists may produce a beneficial effect on PH acting through vasodilatation, inhibition of vascular remodeling, and prevention of RV dysfunction, the hallmarks of PH. To answer this hypothesis, we designed an experimental large-animal study (porcine model of PH by pulmonary vein banding) and ex vivo human experiments with the following objectives: (1) To evaluate the acute hemodynamic effect of β3AR agonists in chronic PH; (2) To evaluate the long-term effect of β3AR agonists on pulmonary hemodynamics, RV performance, and pulmonary vascular remodeling in chronic PH; and (3) To explore the expression of β3AR mRNA and the vasodilator response of β3AR agonists in human pulmonary arteries. This is the first study of a broad project aimed to evaluate the clinical relevance of this potential new therapy (current study) and the mechanisms of action (ongoing work).

## Methods

### Large-animal study design and experimental model

Experimental animal procedures were performed in a total of 34 castrated-male Large-White pigs. The study was approved by the Institutional Animal Research Committee and carried out in compliance with the *Guide for the Care and Use of Laboratory Animals*. Before any procedure, anesthesia was induced by intramuscular injection of ketamine (20 mg/kg), xylazine (2 mg/kg), and midazolam (0.5 mg/kg). Buprenorphine (0.3 mg/kg) was used for analgesia and animals were intubated.

#### Pig model of chronic PH

A model of chronic post-capillary PH was generated by surgical banding of the main pulmonary vein (venous confluent arising from the junction of both inferior pulmonary veins) through a small thoracotomy in 4 week-old piglets (weigh ≈10 kg), as previously described [28]. This procedure has demonstrated to generate progressive chronic PH that becomes stable from the third month [28], associated with typical PH changes on pathology.

##### Proof of concept of β3AR agonists in chronic PH

As a proof of concept in chronic PH, 8 pigs (3-month old and weighing ≈30 kg) with previous pulmonary vein banding and confirmed chronic PH were used to evaluate the acute hemodynamic effect of β3AR agonists. For this aim, free-breathing pigs on room air received intravenously a dose of 5 µg/kg of BRL37344 under right heart catheterization, invasive systemic blood pressure (SBP) and electrocardiographic monitoring. Complete hemodynamic evaluation was repeated after 20 min.

##### Long-term β3AR agonist treatment in chronic PH

To evaluate the effect of long-term therapy with β3AR agonists in chronic PH, two different experiments testing different β3AR selective agonists were conducted: (1) continuous intravenous treatment with the β3AR agonist BRL37344 (R*,R*)-(±)-4-[2-[(2-(3-Chlorophenyl)-2-hydroxyethyl) amino] propyl] phenoxyacetic acid, sodium salt) and (2) oral β3AR agonist mirabegron (Myrbetriq^®^, Astellas Pharma US, Northbrook, IL, USA). An additional arm using nebivolol (Lobivon^®^, Menarini, Luxembourg), a β1AR antagonist and β3AR agonist, was included in the second experiment to evaluate the effect of β3AR agonism with simultaneous β1AR blockade in chronic PH.

On the first long-term experiment, 8 pigs (4-month old and weighing ≈45 kg) with chronic PH were blindly randomized to receive a continuous intravenous infusion of BRL37344 or vehicle (saline) using osmotic pumps (Alzet^®^, DURECT Corp., Cupertino, CA, USA). For this purpose, we used 2 mL osmotic pumps with a pre-specified delivery rate of 5 µL/h during 14 days, and equipped with a polyethylene infusion catheter (polyethylene tubing PE-60). Pumps contained either a dose of 10 µg/kg/day of BRL37344 dissolved in saline or vehicle (saline) and were surgically implanted with the catheter inserted in the right external jugular vein. Right heart catheterization and cardiac magnetic resonance (CMR) imaging were performed immediately before pump insertion and 14 days later.

For the second long-term experiment, another 18 pigs (4-month old and weighing ≈45 kg) with chronic PH were blindly randomized to receive oral treatment with mirabegron (100 mg/day; *n* = 6) or nebivolol (5 mg/day; *n* = 6) administered within an apple or vehicle (only the apple; *n* = 6) for 14 days. Similarly, right heart catheterization and CMR imaging were performed immediately before initiation of treatment and 14 days later. In both experiments, only a veterinarian not involved in the study knew treatment allocation.

### Invasive hemodynamic assessment

SBP was monitored with a femoral arterial cannula (Arrow, Reading, PA, USA). Right heart catheterization was performed using a Swan-Ganz catheter (Braun, Kronberg, Germany) inserted through the femoral vein and positioned under fluoroscopy to measure PAP and cardiac output as assessed by the thermodilution method. PVR was calculated as the difference between mean PAP and left ventricular end-diastolic pressure quantified with a pigtail catheter divided by the cardiac output in Wood units. Cardiac output and PVR were indexed by body surface area estimated by the Brody’s formula [4].

### CMR studies

All CMR studies were performed with a 3.0 T magnet (Achieva-Tx, Philips Medical Systems, The Netherlands), equipped with a 32-channel cardiac phased-array surface coil and retrospective electrocardiographic gating during spontaneous ventilation. Steady-state free precession cine sequences were acquired in 10–15 contiguous short axis slices covering both ventricles from base to apex with 30 cardiac phases each for the evaluation of ventricular volumes and function. Two-dimensional flow imaging (phase-contrast) was performed perpendicular to the main pulmonary artery with a velocity-encoded gradient echo sequence using the minimum upper velocity limit without signal aliasing, as previously described [13].

Analysis of CMR acquisitions was performed using specialized software (Extended MR Workspace^®^, Philips Healthcare, The Netherlands). Observers were blinded to the study arm, experimental allocation (β3AR agonist/vehicle) and hemodynamic measurements. On cine images, biventricular endocardial contours were manually traced in end-diastole and end-systole and Simpson’s method was used to calculate volumes and ejection fraction. RV trabeculations were included within the blood pool and the interventricular septum was adjudicated to the left ventricular mass. Similarly, the inner contour of the main pulmonary artery was outlined in each cardiac phase to quantify the minimum and maximum areas, average pulmonary artery velocity, and RV stroke volume. Ventricular volumes and masses and pulmonary artery areas were similarly adjusted to body surface area.

### Histological studies

#### Pig samples

At the completion of the study, animals with chronic PH randomized to the oral β3AR agonist mirabegron or vehicle were euthanized with a lethal injection of pentobarbital sodium and the lung parenchyma was excised for histological evaluation. Expression of P27 and Ki67 was compared between groups to assess differences in cell proliferation in pulmonary arteries within lung parenchyma.

##### Western blot for P27 expression

Randomly chosen lower lobe pulmonary parenchyma portions were snap frozen in liquid nitrogen and stored at −80 °C until used. For protein isolation, samples were homogenized using a radio-immunoprecipitation assay lysis buffer (150 mmol/L NaCl, 1.0 % IGEPAL, 0.5 % sodium deoxycholate, 0.1 % SDS, 50 mmol/L Tris, pH 8.0) supplemented with protease/phosphatase inhibitors. After quantification (Pierce BCA Protein Assay Kit, Pierce Biotechnology, Rockford, IL, USA), 50 µg of proteins were loaded in 15 % sodium dodecyl sulfate polyacrylamide gels. Proteins were transferred into a polyvinylidene fluoride membrane following electrophoresis and incubated with the primary specific antibody for p27 (BD Biosciences, Santa Fe, CA, USA) overnight in Tris Buffer Saline + 0.1 % Tween + 5 % Bovine Serum Albumin. Total ERK 1-2 (Cell Signaling, Danver, MA, USA) protein was used as loading control. Blots were developed by chemiluminescence after HRP-secondary antibody, 1-hour incubation using Luminata Forte substrate (EMD Millipore, Billerica, MA, USA). Intensity of the bands was analyzed using the ImageJ 6.0 software (NIH, Bethesda, MD, USA) and the ratio between p27 and total ERK 1-2 protein was calculated in densitometry units.

##### Immunohistochemical staining for Ki67 expression

Randomly chosen lower lobe pulmonary parenchyma portions were fixed in 4 % paraformaldehyde for 48 h, dehydrated and embedded in paraffin. Specific staining for Ki67 (Master Diagnostica, Granada, Spain) and haematoxylin was performed in 4 µm sections. An average size of 200 mm^2^ for each parenchyma was digitalized using the NanoZoomer 20RS scanner (Hamamatsu Photonics K.K., Hamamatsu, Japan) at a magnification of 40x. The quantification of the positive Ki67 nuclei in the wall of the arteries was performed in the digitalized images using the NanoZoomer digital Pathology viewer (Hamamatsu Photonics K.K., Hamamatsu, Japan). A number of 24 arteries with a diameter between 30 and 100 µm were quantified per subject and the results represented as median (interquartile range) of Ki67 positive nuclei per artery (wall) and also adjusted by the arterial diameter.

#### Human samples

##### Human pulmonary artery smooth muscle cell cultures

Human pulmonary artery smooth muscle cells were purchased from ScienCell Research Laboratories (Carlsbad, CA, US) and cultured in a specific medium (Sciencell No. 1101, Carlsbad, CA, US) supplemented with 2 % fetal bovine serum 100 U/mL penicillin, 100 µg/mL streptomycin, and 1 % smooth muscle cells growth supplement (Sciencell No. 1152, Carlsbad, CA, USA) in a humidified incubator with a constant supply of 5 % CO_2_ at 37 °C to be used for experiments at passages 3–7.

Human pulmonary artery smooth muscle cells were seeded in gelatin 0.1 % coated 48-well plates (same number per well) and starved in medium with 0.4 % FBS for 24 h and then incubated under either normoxic or hypoxic (3 % O_2_, 5 % CO_2_ at 37 °C) conditions for 72 h. For treatment, cells were co-incubated with BRL37344 (0.001 μM) alone or associated with NG-nitro-l-arginine methyl ester (L-NAME 10 mM, Sigma-Aldrich Co. LLC.) during the hypoxia exposure. Human pulmonary artery smooth muscle cells proliferation was determined by cell counting using flow cytometry. All experiments were performed in triplicate.

##### RNA isolation and cDNA synthesis

Collection of human main pulmonary arteries and lung samples was carried out at Hospital Clínic (Barcelona). The Ethics Committee of this Institution approved the research protocol and all patients gave informed consent. Main pulmonary artery samples were obtained from patients undergoing elective heart transplantation (*n* = 10, urgent cases were excluded). No patient received sympathomimetic drugs in days before transplantation. Main pulmonary artery tissue was homogenized and RNA was extracted using RNeasy Mini Kit (Qiagen, Valencia, CA, USA) according to manufacturer’s instructions.

RNA was quantified by measurement of optical density at 260 nm using the spectrophotometer Nanodrop ND-1000 (ThermoScientific, Wilmington, DE, USA) and frozen at −80 °C until their usage. A total of 1 μg of RNA were taken to perform Reverse Transcriptase using Illustra Ready-to-go RT-PCR Beads kit (GE Healthcare Bio-sciences, Pittsburgh, PA, USA) according to manufacturer’s instructions. A final volume of 50 µL was incubated at 42 °C for 30 min followed by 5 min at 95 °C.

##### Real-time quantitative RT-PCR

G0ene expressions assay was performed using the ABI PRISM^®^ 7900HT FAST Real-Time PCR System (Life Technologies, Carlsbad, CA, USA). A 10 µL of real-time PCR reaction Power SYBR Green PCR Master Mix (Life Technologies, Carlsbad, CA, USA) was used following manufacturer’s instructions. Sequences of the primers were as follow: for hβ3AR, 5′-ACCTTCCTCTTCTCGTGATGC-3′ (sense) and 5′-GGCGGAGACTCCTCGGG-3′ (antisense); and for the housekeeping gene 18S, 5′-AGTTGGTGGAGCGATTTG-3′ (sense) and 5′-TTGCTCAATCTCGGTGG-3′. They were designed using human GenBank and Basic Local Alignment Search Tool (BLAST; NCBI). The following temperature–time profile was used to perform the real-time PCR: pre-incubation at 95 °C for 10 min, 40 cycles for amplification of the targets and controls cDNA consisting of denaturation at 95 °C for 15 s; annealing and extension at 60 °C for 60 s. Specific amplification was confirmed by the presence of a single peak in the melting curve plots. PCR products were electrophoretically separated in a 3 % agarose gel to confirm the 105 base pair amplicon product.

### Organ bath studies

Organ bath studies were performed to evaluate the vasodilator effect of BRL37344 in small human pulmonary arteries accordingly with previous methodology [27]. Briefly lung samples were obtained from patients (*n* = 10) undergoing lobectomy or pneumonectomy after informed consent. In the laboratory, 2 mm-diameter arteries were cleaned of fat and connective tissue and cut into rings (3 mm long). Rings were suspended on stainless-steel wires in an organ bath containing Krebs solution. The temperature of the bath was maintained at 37 ± 0.5 °C and the Krebs solution was continuously oxygenated with a 21 %O_2_, 5 % CO_2_ and rest nitrogen mixture. Arterial rings were progressively stretched to a stable resting tension of 1.75 g. Isometric tension was recorded using a force displacement transducer (PanLab, Barcelona, Spain) and displayed on a computer. After a 2 h equilibration period, with the Krebs solution being changed every 15 min, the rings were contracted with norepinephrine (1 × 10^−7^–1 × 10^−6^ mol/L). Once the contraction reached a plateau, cumulative concentration–response curves to BRL37344 (1 × 10^−8^–1 × 10^−4^mol/L) were constructed. The relaxation produced by each concentration was measured after a steady-state was reached. Values were expressed as the percentage change in the maximal tension of rings after addition of norepinephrine.

### Determination of BRL37344, mirabegron and nebivolol in plasma

Plasma concentrations of BRL37344, mirabegron and nebivolol were determined by LC–MS/MS method in three different pigs 4 and 6 h after implantation of BRL37344 pump or first mirabegron or nebivolol dose, and everyday before daily dose administration during 1 week.

At scheduled time points, 2 mL blood samples were taken via venipuncture and collected into tubes containing heparin as an anticoagulant. Following blood sampling, sample tubes were centrifuged at 1500 rpm and 4 °C during 20 min. The separated plasma was immediately stored at −70 °C till analysis. Before analysis, ammonium acetate (20 mmol/L) and acetic acid were added to the plasma to get a pH around 5. Then the samples were lyophilized. Lyophilized plasma was re-suspended in methanol (same amount) and centrifuged at 13,400 rpm for 15 min. The upper organic layer was injected into the LC–MS/MS system.

### Statistical analysis

Continuous variables are expressed as median (interquartile range), otherwise specified. Given the small sample sizes, all variables were considered as non-normally distributed. Mann–Whitney *U* test was used to compare baseline continuous variables between groups, and to evaluate whether the change in the outcome variables from pre to post-treatment differed between two groups. Differences were considered statistically significant at *p* value <0.05. Analyses were performed with SPSS version 20 (IBM Corp., Armonk, New York).

## Results

### Effect of β3AR agonists on hemodynamics and RV performance in experimental chronic PH

*Proof of concept study* In chronic PH [*n* = 8, mean PAP 37.5 (17.0), PVRI 7.6 (7.7)], a single bolus of BRL37344 (5 µg/kg) caused, 20 min after intervention, a significant reduction in PAP and PVRI associated with an increase in CI (Table 1).

**Table 1:** Acute hemodynamic effect of β3AR agonist treatment using BRL37344 in chronic PH

	Baseline (*N* = 8)	20 min after BRL37344 (*N* = 8)	Change	*p*
Weight (kg)	33.0 (17.5)	NA	NA	NA
SpO_2_ (%)	90.5 (2.0)	93.0 (3.0)	3.0 (2.75)	**0.011**
HR (bpm)	91.0 (39.0)	93.0 (30.0)	−6.0 (14.0)	0.199
Systolic BP (mmHg)	113.5 (30.0)	105.0 (31.0)	−8.0 (12.75)	0.058
Diastolic BP (mmHg)	70.5 (17.0)	63.0 (3.0)	−9.0 (18.0)	0.063
Mean BP (mmHg)	90.5 (23.0)	80.0 (15.0)	10.0 (8.75)	0.109
Systolic PAP (mmHg)	51.0 (20.0)	38.0 (15.0)	−12.0 (13.25)	**0.002**
Diastolic PAP (mmHg)	27.0 (15.0)	17.0 (12.0)	−6.0 (5.5)	**<0.001**
Mean PAP (mmHg)	37.5 (17.0)	26.0 (12.0)	−9.0 (6.0)	**<0.001**
Cardiac index (L/min/m^2^)	4.0 (1.2)	4.7 (1.1)	0.33 (1.48)	**0.018**
PVRI (WU/m^2^)	7.6 (7.7)	3.9 (6.9)	−2.5 (3.6)	**0.006**

Values are expressed as median (interquartile range)

*SpO*_*2*_ oxygen saturation by pulse oximetry, *HR* heart rate, *bpm* beats per minute, *BP* blood pressure, *PAP* pulmonary artery pressure, *PVRI* pulmonary vascular resistance index, *WU* wood units

**Fig. 1:**
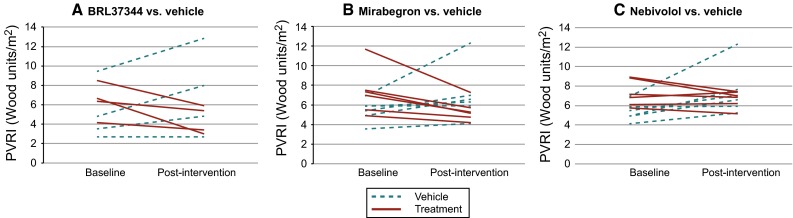
Effect of β3AR stimulation vs. vehicle on PVRI in chronic PH. Individual data are shown in the *first row* whereas *boxplots* by intervention groups are shown in the *second row*

*Long-term studies* In the first experiment [*n* = 8, mean PAP 32.5 (8.8), PVRI 5.7 (4.0)], animals randomized to long-term intravenous therapy with the β3AR agonist BRL37344 (10 µg/kg/day for 14 days) showed a significant reduction in PVRI and an increase in CI compared with vehicle (Table 2; Fig. 1a).

**Table 2:** Long-term effect of continuous intravenous β3AR treatment with BRL37344 on hemodynamics in chronic PH

	Baseline		Post-treatment		Change 14 days after intervention		
	Vehicle (*N* = 4)	BRL37344 (*N* = 4)	Vehicle (*N* = 4)	BRL37344 (*N* = 4)	Vehicle (*N* = 4)	BRL37344 (*N* = 4)	*p*
Weight (kg)	43.8 (20.6)	50.8 (19.3)	57.8 (22.8)	64.0 (26.9)	13.5 (5.1)	12.8 (8.1)	0.644
SpO_2_ (%)	95.0 (3.0)	92.5 (9.0)	92.0 (4.0)	88.5 (7.0)	−3.5 (4.0)	−1.5 (9.0)	0.715
HR (bpm)	73.0 (15.0)	72.5 (14.0)	77.5 (47.0)	84.0 (23.0)	0.5 (53.7)	15.0 (18.3)	0.535
Systolic BP (mmHg)	113.5 (21.0)	120.0 (21.0)	118.0 (22.0)	113.5 (11.0)	9.5 (10.8)	−4.5 (11.5)	0.034
Diastolic BP (mmHg)	76.0 (17.0)	74.5 (6.0)	71.0 (28.0)	74.5 (8.0)	−1.5 (16.3)	4.0 (7.8)	0.773
Mean BP (mmHg)	93.0 (20.0)	94.0 (13.0)	93.5 (24.0)	91.0 (8.0)	5.0 (12.8)	−0.5 (9.0)	0.219
Systolic PAP (mmHg)	38.0 (25.5)	46.5 (13.5)	45.8 (20.8)	40.8 (10.0)	3.3 (11.4)	−4.5 (12.0)	0.167
Diastolic PAP (mmHg)	23.0 (8.3)	25.5 (6.3)	30.0 (16.5)	25.8 (8.4)	6.0 (13.3)	1.8 (11.4)	0.501
Mean PAP (mmHg)	29.5 (13.0)	36.0 (8.0)	38.3 (18.9)	32.2 (7.9)	5.8 (11.9)	−0.8 (9.9)	0.185
Cardiac index (L/min/m^2^)	4.4 (1.0)	4.5 (1.5)	4.4 (1.4)	5.3 (2.0)	−0.4 (1.5)	0.9 (0.6)	**0.042**
LV End-diastolic pressure (mmHg)	8.5 (3.0)	6.5 (3.0)	9.5 (8.0)	9.0 (2.0)	1.0 (5.8)	2.0 (2.8)	0.775
PVRI (WU/m^2^)	4.9 (4.2)	6.5 (3.6)	6.4 (8.1)	4.4 (2.7)	1.5 (4.1)	−2.0 (2.7)	**0.040**

Values are expressed as median (interquartile range)

*SpO*_*2*_ oxygen saturation by pulse oximetry, *HR* heart rate, *bpm* beats per minute, *BP* blood pressure, *PAP* pulmonary artery pressure, *PVRI* pulmonary vascular resistance index, *WU* wood units

Comparable results were observed in the second experiment [*N* = 18, mean PAP 37.5 (8.3) mmHg, PVRI 6.4 (2.1) Wood units/m^2^]. Animals randomized to long-term oral treatment with the oral β3AR agonist mirabegron (100 mg/day for 14 days) showed a significant reduction in PVRI and an increase in CI compared with vehicle (Table 3; Fig. 1b). Finally, animals randomized to nebivolol (oral β3AR agonist and β1AR antagonist) showed a significant reduction in PVRI as well, without significant change in cardiac index (Table 3; Fig. 1c).

**Table 3:** Long-term effect of oral β3AR agonist treatment with mirabegron or nebivolol on hemodynamics in chronic PH

	Baseline			Post-treatment			Change				
	Vehicle (*N* = 6)	Mirabegron (*N* = 6)	Nebivolol (*N* = 6)	Vehicle (*N* = 6)	Mirabegron (*N* = 6)	Nebivolol (*N* = 6)	Vehicle (*N* = 6)	Mirabegron (*N* = 6)	Nebivolol (*N* = 6)	*p* ^1^	*p* ^2^
Weight (kg)	47.7 (12.8)	39.5 (18.6)	48.2 (13.5)	54.0 (14.8)	48.8 (19.8)	54.5 (14.8)	8.0 (3.6)	12.0 (5.0)	10.5 (3.0)	0.180	0.180
SpO_2_ (%)	88.0 (10.0)	89.0 (13.0)	84.5 (9.0)	88.5 (5.0)	88.5 (13.0)	90.0 (8.0)	−0.5 (11.0)	3.0 (10.2)	5.5 (5.8)	0.310	**0.041**
HR (bpm)	69.0 (21.0)	83.0 (19.0)	86.0 (9.0)	76.0 (16.0)	97.0 (12.0)	83.0 (18.0)	12.0 (40.2)	15.0 (23.2)	−6.0 (13.3)	0.394	0.394
Systolic BP (mmHg)	120.5 (10.0)	116.5 (11.0)	128.5 (34.0)	131.0 (15.0)	118.5 (15.0)	124.5 (17.0)	10.0 (19.3)	6.5 (12.3)	5.0 (27.0)	0.485	0.937
Diastolic BP (mmHg)	81.5 (17.0)	74.0 (11.0)	88.0 (15.0)	87.5 (13.0)	80.0 (16.0)	80.5 (13.0)	9.5 (20.8)	5.0 (10.3)	−2.5 (12.8)	0.589	0.180
Mean BP (mmHg)	96.5 (16.0)	91.5 (9.0)	104.0 (20.0)	106.0 (17.0)	96.5 (17.0)	97.0 (16.0)	12.0 (23.0)	5.5 (12.5)	−3.5 (17.8)	0.310	0.180
Systolic PAP (mmHg)	45.5 (12.3)	48.5 (14.5)	55.0 (18.3)	52.0 (23.8)	48.5 (14.8)	56.5 (16.5)	8.5 (24.0)	0.0 (10.2)	−2.0 (7.5)	0.180	0.093
Diastolic PAP (mmHg)	27.0 (7.0)	26.5 (8.5)	33.0 (9.5)	28.5 (17.0)	29.0 (13.8)	29.5 (6.3)	5.5 (20.5)	4.0 (8.0)	−3.5 (5.8)	0.589	0.065
Mean PAP (mmHg)	35.0 (9.3)	36.5 (10.5)	42.0 (13.3)	38.5 (15.0)	37.5 (13.3)	42.0 (10.3)	6.5 (18.8)	0.5 (5.0)	−2.0 (8.5)	0.240	0.093
Cardiac index (L/min/m^2^)	4.8 (1.1)	4.4 (0.4)	4.5 (1.0)	4.7 (0.9)	5.4 (0.8)	4.9 (0.7)	−0.3 (1.6)	1.1 (1.1)	0.2 (0.3)	**0.026**	0.394
LV End-diastolic pressure (mmHg)	8.5 (5.0)	7.5 (6.0)	8.5 (2.0)	8.5 (3.9)	9.0 (3.0)	9.0 (2.0)	0.5 (2.8)	0.5 (3.2)	0.0 (4.0)	0.589	0.937
PVRI (WU/m^2^)	5.2 (1.4)	7.1 (3.1)	7.0 (2.8)	6.8 (3.1)	5.1 (1.8)	6.9 (1.3)	1.6 (2.6)	−1.8 (1.7)	−0.5 (1.9)	**0.002**	**0.009**

Values are expressed as median (interquartile range)

*p* ^*1*^ Mirabegron vs. placebo, *p* ^*2*^ Nebivolol vs. placebo, *SpO*_*2*_ oxygen saturation by pulse oximetry, *HR* heart rate, *bpm* beats per minute, *BP* blood pressure, *PAP* pulmonary artery pressure, *PVRI* pulmonary vascular resistance index, *WU* wood units

Chronic therapy with selective β3AR agonist [BRL37344 (*n* = 4) or mirabegron (*n* = 6)] was associated with improved RV performance on CMR evaluation as compared with placebo (*n* = 10). Two weeks after treatment, there were significant differences in the change in RV systolic volume and RV ejection fraction (Table 4). In addition, a significant increase in average pulmonary artery velocity in the β3AR-treated group was observed, a surrogate non-invasive measurement of PVR [13].

**Table 4:** Long-term effect of selective β3AR agonist treatment with BRL37344 or mirabegron on CMR parameters in chronic PH

	Baseline		Post-treatment		Change		
	Vehicle (*N* = 10)	β3AR agonist (*N* = 10)	Vehicle (*N* = 10)	β3AR agonist (*N* = 10)	Vehicle (*N* = 10)	β3AR agonist (*N* = 10)	*p*
Weight (kg)	47.2 (12.3)	42.7 (18.6)	57.3 (13.6)	54.0 (24.3)	10.2 (5.7)	12.0 (5.4)	0.481
HR (bpm)	67.5 (33.0)	79.0 (28.0)	73.0 (22.0)	85.5 (14.0)	4.5 (37.5)	15.0 (18.2)	0.631
RV end-diastolic volume index (ml/m^2^)	99.3 (20.6)	104.0 (15.5)	101.4 (16.5)	92.2 (16.8)	3.6 (18.2)	−4.0 (26.1)	0.143
RV end-systolic volume index (ml/m^2^)	37.9 (18.4)	46.6 (16.1)	43.7 (5.3)	39.7 (7.0)	6.5 (15.7)	−5.4 (9.8)	**0.009**
LV end-diastolic volume index (ml/m^2^)	97.1 (9.5)	90.3 (17.7)	93.2 (11.8)	93.3 (17.3)	−1.4 (16.4)	3.4 (11.3)	0.436
LV end-systolic volume index (ml/m^2^)	35.5 (11.0)	36.2 (9.0)	37.5 (8.7)	37.2 (11.6)	1.0 (7.7)	1.0 (7.7)	0.971
RV mass index (g/m^2^)	28.1 (8.1)	26.6 (7.6)	28.1 (8.2)	27.6 (3.6)	0.0 (8.3)	1.9 (8.3)	0.796
LV mass index (g/m^2^)	58.8 (11.4)	52.6 (9.1)	62.1 (7.0)	60.1 (8.3)	−2.1 (17.6)	7.3 (15.8)	0.247
RV ejection fraction (%)	61.9 (13.0)	52.0 (6.4)	56.4 (5.6)	58.4 (7.8)	−3.6 (9.3)	5.0 (5.2)	**0.007**
LV ejection fraction (%)	63.5 (4.2)	59.8 (6.1)	61.9 (6.3)	61.2 (6.39	−1.0 (4.6)	0.6 (6.3)	0.280
PA average velocity (m/s)	11.0 (3.3)	10.5 (4.9)	11.7 (3.7)	12.3 (3.2)	0.9 (2.7)	1.9 (2.5)	**0.019**
PA maximal area (cm^2^)	7.0 (1.7)	7.8 (3.6)	7.0 (2.9)	7.5 (2.9)	−0.3 (1.7)	−0.6 (1.5)	0.089
PA minimal area (cm^2^)	4.9 (2.2)	5.9 (3.2)	5.3 (2.4)	5.7 (2.4)	0.4 (1.5)	−0.3 (2.1)	0.075

Values are expressed as median (interquartile range)

*HR* heart rate, *bpm* beats per minute, *RV* right ventricular, *LV* left ventricular, *PA* pulmonary artery, *p* ^*1*^ *p* value from Student’s *t* test or Mann–Whitney *U* test, as adequate; *p* ^*2*^ *p* value for the time*group interaction term in the repeated measures ANOVA or Mann–Whitney *U* test of changes, as adequate

### Effect of β3AR agonists on p27 and Ki67 expression in lungs from chronic PH pigs

**Fig. 2:**
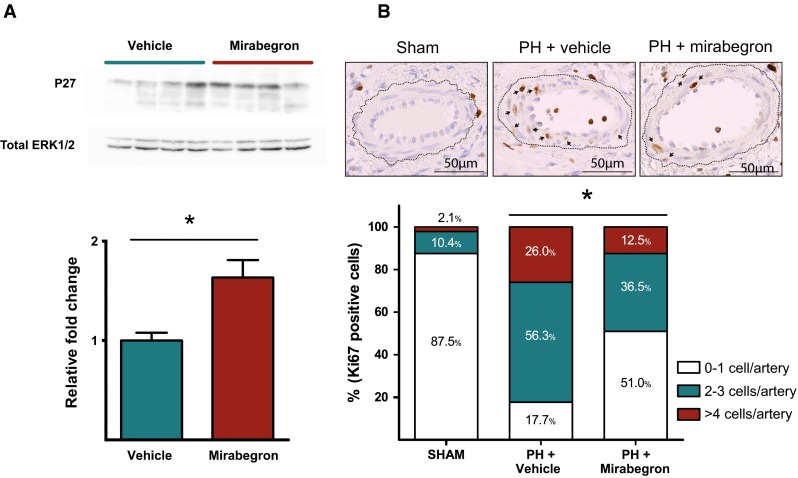
Protein expression related with pulmonary cellular proliferation. **a** Western blot for the P27 protein in the lung parenchyma from pigs with chronic PH receiving vehicle (*N* = 4) or mirabegron (*N* = 4) for 14 days. The densitometric analysis of P27 normalized to total ERK 1/2 is shown below. **b** Representative immunohistochemical pictures for Ki67 staining in pulmonary arteries within the lung parenchyma from sham-operated controls, pigs with chronic PH treated with vehicle and pigs with chronic PH treated with mirabegron. *Brown staining* indicates Ki67-positive cells. *Arrows* indicate Ki67-positive cells within the arterial wall. The *frequency bar chart* shown below represents the percentage of pulmonary arteries with Ki67 positive cells categorized in three groups (0–1 positive cell/artery, 2–3 positive cells/artery and >4 positive cells per artery) by group. *Statistically significant differences

Pigs with chronic PH treated with the oral β3AR agonist mirabegron showed a significant increase in p27 protein levels as compared with the controls (Fig. 2a). The density of Ki67 positive cells was lower in pulmonary arteries from PH pigs treated with mirabegron compared with those treated with the vehicle alone [3 (2) vs. 1 (2) cells/artery, *p* < 0.01). Similar results were obtained after adjusting with the arterial diameter [3.5 (2.4) vs. 2.2 (2.6) cells/artery/µm × 10^2^, *p* < 0.01) or whether analyzed as a categorical variable (Fig. 2b).

### Effect of β3AR agonist on human hypoxia-induced pulmonary artery smooth muscle cell proliferation

**Fig. 3:**
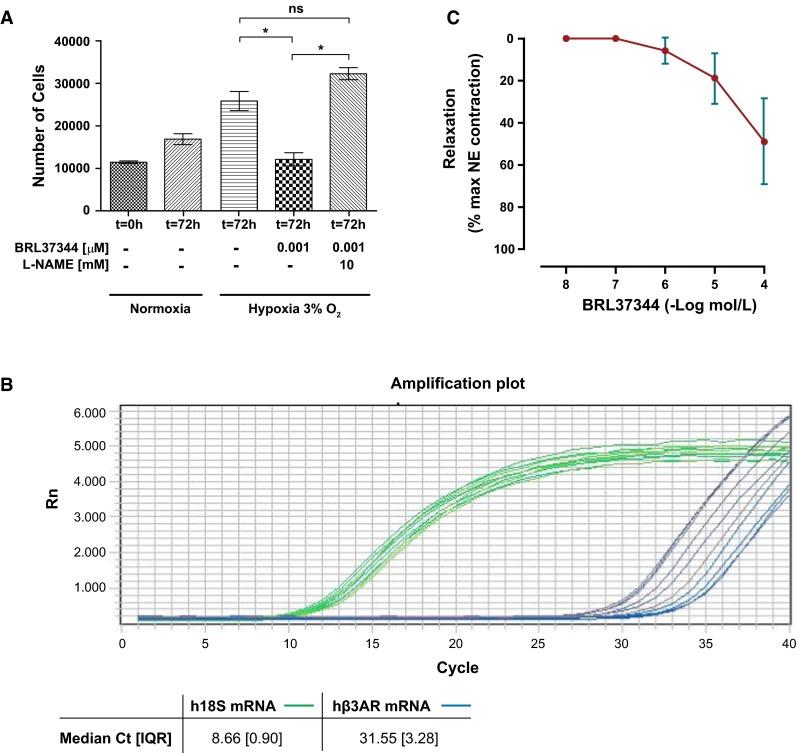
Ex vivo experiments in human pulmonary artery smooth muscle cells and pulmonary arteries. **a** Effect of BRL37344 on hypoxia-induced proliferation of human pulmonary artery smooth muscle cell proliferation. Cell proliferation was measured by cytometry after 72 h of hypoxia exposure. Data represent mean value and SD of three independent experiments. *p < 0.05; *NS* non significant. **b** Human b3AR mRNA expression in human pulmonary arteries (*N* = 10). Amplification curves for hb3AR (*blue*) and 18S (*green*) mRNA expression. The values of the median Ct (IQR) are shown in the table. **c** Vasodilator effect of BRL37344 on human pulmonary arteries (*N* = 10). Relaxation of small pulmonary arteries to cumulative concentrations of BRL37344, expressed as % of contraction to norepinephrine (NE). Maximal NE response was 18,495 (3420) mN. Values are mean ± SD

Hypoxia (72 h, 3 %) induced increased proliferation of human pulmonary artery smooth muscle cells that was inhibited by BRL37344. Co-incubation with L-NAME abolished the inhibitory effect of BRL37344 over proliferation (Fig. 3a).

### Detection of β3AR mRNA expression in human pulmonary arteries and vasodilator effect of BRL37344

We detected mRNA expression of hβ3AR in all human pulmonary arteries by qPCR. The amplification curves for the human hβ3AR and the human 18s are shown in Fig. 3b.

BRL37344 induced a dose-dependent relaxation in norepinephrin-precontracted human pulmonary artery rings (maximal relaxation of 51.0 ± 7.7 % achieved at 10^−4^ M, with maximal NE response of 18,495 (3420) mN.) (Fig. 3c).

### Plasma concentrations of BRL37344, mirabegron and nebivolol

Plasma concentration of BRL37344 was 1.37 and 5.15 ng/mL at 4 and 6 h after pump implantation, respectively. Levels remained stable around 1 ng/mL (0.28–0.97) on daily analyses during 7 days.

Plasma concentration of mirabegron was 0.4 and 2.82 ng/mL at 4 and 6 h after administration. Levels remained stable around 1 ng/mL (0.7–1.5) on daily pre-dose analyses during 7 days.

Plasma concentration of nebivolol was 0.14 and 0.08 ng/mL at 4 and 6 h after administration. Levels remained stable ranging between 0.05 and 0.06 ng/mL on daily pre-dose analyses during 7 days.

## Discussion

This study represents the first evidence of the beneficial effects of β3AR agonists in PH. The main findings of the study are: (1) Treatment with β3AR agonists reduces PVRI and improves RV performance in an experimental large-animal translational model of chronic PH; (2) In lung tissue, long-term therapy with a β3AR agonist is associated with changes in protein expression suggestive of attenuated vascular proliferation; and (3) β3AR is expressed in human pulmonary arteries and β3AR agonist administration inhibits human pulmonary artery smooth muscle cell proliferation by a nitric oxide dependent mechanism and produces vasodilatation ex vivo.

PH is a prevalent and serious condition characterized by increased PAP and PVR and progressive RV dysfunction. In the present study, we demonstrated that treatment with β3AR agonists reduces PVRI and increases CI in translational experimental large-animal models of acute and chronic PH, as assessed with right heart catheterization (the gold-standard technique). No significant changes were observed in SBP and HR, although the study may have been underpowered to detect differences in HR, parameter with high individual variability. A reduction in PVR with increased cardiac output demonstrated a vasodilator effect. The increase in cardiac output might be secondary to a reduction in afterload or a direct effect over the damaged RV, as observed in the LV [5]. It has been repeatedly shown that PVR provides a more consistent prognostic value than mean PAP in PH of different etiologies [6, 32]. Additionally, in advanced stages of chronic PH, progressive RV failure can be accompanied by a decrease in PAP, whereas PVR continues to increase. In the specific case of PH associated with end-stage left heart disease, PVR is of paramount importance when deciding whether a patient might still be a candidate for heart transplantation [22].

There are two major classes of high-affinity β3ARs agonists, the phenylethanolamines that comprises BRL37344 and others, and the aryloxypropanolamines where mirabegron is included. Unlike BRL37344, mirabegron is available as oral tablet with extended release. Thereby, the second experiment of long-term therapy using mirabegron was performed to make the project and its results more translational since it would be potentially easier to treat patients with an oral β3AR agonist, which is, in addition, already approved for human use to treat hyperactive bladder syndrome (50 mg/day) [7]. The pharmacokinetic profile of mirabegron after single and multiple oral doses has been already reported in several cohorts of Caucasian [11, 19] and Asian adult subjects [18]. All have demonstrated that mirabegron plasma concentrations peaked at 3–5 h and achieved steady-state within 7 days of once daily administration, similar as found in our experimental model.

All hemodynamic effects were similar for the two different selective β3AR agonists tested, which reinforces the results obtained and suggests that it is the target activation which is important and not the drug class *per se*. As expected, the effect of nebivolol was different because it possesses a β3AR agonist effect with a β1AR antagonist activity. Derived from its negative inotropic β1AR blocking effect, a neutral effect on cardiac index was observed, while a significant reduction of PVR was maintained. This result is consistent with recent data pointing to nebivolol as a potential beneficial therapy for PH [29, 31]. Additionally, this third arm helped us exclude that the effect over PVR was derived from non-selective β1AR stimulation. However, although several studies have shown that nebivolol vasodilates in vitro [9] and in vivo [2] through an agonist effect on endothelial β3ARs, other studies have failed to demonstrate binding to β3AR [12], so results obtained with nebivolol should be considered with caution since the degree they are produced by β3AR stimulation is uncertain.

The concentration of BRL37344 in plasma was around 3 ng/mL during first hours, reaching a steady-state concentration around 1 ng/mL. Considering that the molecular weight of BRL37344 is 385.8 g/mol, the concentration achieved in our animals fell inside the concentration range that showed affectivity in vitro in human arteries (10^−6^ M equals 0.38 ppb and 10^−5^ M equals 3.856 ppb). Based on studies evaluating selective signal transduction in the β3AR [3], we consider that these drug levels mainly acted through β3AR activation, as BRL37344 Ki values are 287 and 1750 nM for β3 and β1 receptors, respectively. Similarly occured with mirabegron (EC50 of 22.4 (12.6–36.3) for β3 vs. >10,000 for β1 [34].

Hemodynamic changes with long-term β3AR agonist therapy were associated with an improvement in RV performance assessed by CMR, the gold-standard imaging technique, as evidenced by a decrease in RV end-systolic volume and an improvement in RV ejection fraction. This is relevant due to the prognostic importance of RV performance in chronic PH.

The downstream pathway activated by β3AR agonists is known to include nitric oxide synthase, nitric oxide-activated guanylate cyclase and guanylate cyclase-cyclic guanylate monophosphate synthesis, and increased cyclic adenosine monophosphate synthesis [30]. Within the pulmonary circulation, cyclic nucleotides are responsible for mediating endothelium-dependent dilation but they also have beneficial effects on pulmonary vascular remodeling and RV function. In fact, in our study, long-term treatment with β3AR agonists in pigs with chronic PH was associated with changes in protein expression associated with a reduced vascular proliferation within the lung parenchyma, suggesting a beneficial protective effect against vascular remodeling. P27 is a key cyclin-dependent kinase inhibitor that blocks the G1 to S-phase transition in cell cycle progression and its role in pulmonary artery smooth muscle cell proliferation in PH has been repeatedly demonstrated [20, 39]. In our study, a decrease in the expression of the cellular proliferation marker Ki67 was correlated with an increase in p27 expression in the lung parenchyma of pigs treated with mirabegron, in agreement with previous PH studies using different therapies [40]. Inhibition of hypoxia-induced proliferation was confirmed in human pulmonary artery smooth muscle cell cultures in vitro. The experiment performed suggested that this inhibitory effect was mediated by nitric oxide.

To the best of our knowledge, our study is the first to report the expression of β3AR mRNA and to evaluate the vasodilator effect of β3AR agonists in human pulmonary arteries. The magnitude of vasodilation observed was consistent with that found in vitro using BRL37344 in human internal mammary arteries [31] or pulmonary arteries from dogs [33].

Most experiments were performed in a translational large-animal model of chronic PH. This model reproduces chronic PH changes in hemodynamics, RV remodeling and pathology [13, 28]. Several novel therapies have demonstrated beneficial effects in pre-clinical studies but have subsequently failed in the clinical arena. This poor translation is in part due to the absence of randomized studies in pre-clinical large-animal models. We have developed our pig model because of its anatomical and physiological similarities to humans. To facilitate the inference of our results to patients, all results were assessed using the same gold-standard methodologies used in clinical practice (right heart catheterization and CMR).

Some limitations should be acknowledged. Our pig model of post-capillary PH may not represent the entire spectrum of patients with PH due to left heart disease, especially because left ventricular function is preserved. However, our study aimed to evaluate the specific effect of β3AR agonists on the pulmonary circulation and the RV since the beneficial effect of β3AR agonists on left ventricular dysfunction has been previously studied. Overt RV dysfunction using this model was observed in few cases (only two animals with baseline RVEF <50 %), thus precluding us to evaluate the effect of the new therapy in the setting of advanced RV dysfunction. Sample sizes were small and therefore, the study might have been underpowered to assess differences in some parameters like BP or HR. The current study focused on the evaluation of the effect of β3AR agonists in a translational animal model of chronic PH. No β3AR antagonist or β2AR antagonist drugs were used, and therefore, we cannot exclude that part of the observed effects using BRL37344 and mirabegron were caused by β2AR stimulation. Further research, on the functional activity of β3ARs in human pulmonary arteries, and the effect of β3AR agonists on them is necessary, including mechanistic studies on the pathways involved. The LC–MS/MS method measured plasma levels of BRL37344 and mirabegron are not bound to plasma proteins. Differences in receptor activation and in protein binding may exist among species. Therefore and specifically, further studies aiming to evaluate the pharmacokinetic profile of the β3AR agonist treatments used in relation with the resultant activation on the target receptor in the pig and human are necessary.

In conclusion, β3AR agonists produced a significant reduction in PVRI and improved RV performance in a translational experimental large-animal model of PH. β3AR agonists emerges as a potential novel approach for treating patients with PH.

## Abbreviations

PVR: Pulmonary vascular resistance
PVRI: Pulmonary vascular resistance index
RV: Right ventricular
β3AR: Beta-3 adrenergic receptor
PH: Pulmonary hypertension
CMR: Cardiac magnetic resonance
PAP: Pulmonary arterial pressure
CI: Cardiac index
SBP: Systemic blood pressure
HR: Heart rate
